# Hepatoprotective Activity of BV-7310, a Proprietary Herbal Formulation of *Phyllanthus niruri*, *Tephrosia purpurea*, *Boerhavia diffusa,* and *Andrographis paniculata*, in Alcohol-Induced HepG2 Cells and Alcohol plus a Haloalkane, CCl_4_, Induced Liver Damage in Rats

**DOI:** 10.1155/2020/6428906

**Published:** 2020-03-25

**Authors:** Debendranath Dey, Sunetra Chaskar, Narendra Bhatt, Deepa Chitre

**Affiliations:** ^1^Bioved Pharmaceuticals, Inc., 1929 OToole Way, San Jose, CA 95131, USA; ^2^Bioved Pharmaceuticals Pvt. Ltd, 5-6/12, Vadgaon Khurd, Pune 411 041, India

## Abstract

Excessive alcohol consumption is a worldwide threat with severe morbidity and mortality. Other than abstinence, there is still no FDA-approved drug for alcoholic liver disease (ALD). Liver is the primary site of ethanol metabolism and hence gets the most damage from excessive drinking. It triggers multiple signalling events including inflammation, leading to an array of hepatic lesions like steatosis, hepatitis, fibrosis, and cirrhosis. Similarly, when medications or xenobiotic compounds are ingested orally, the liver gets the highest exposure of those metabolites, which in turn can cause severe liver toxicity. BV-7310 is a standardized mixture of four Ayurvedic plants, namely, *Phyllanthus niruri, Tephrosia purpurea*, *Boerhavia diffusa,* and *Andrographis paniculata.* In different systems of traditional medicine, each of these plants has been known to have use in gastrointestinal disorders. We wanted to assess the combined effect of these plant extracts on alcohol-induced liver damage. First, we investigated the hepatoprotective activity of BV-7310 against alcohol-induced toxicity in human liver HepG2 cells. Ethanol treatment (120 mM for 48 hours) significantly showed toxicity (around 42%) in these cells, and coincubation with BV-7310 prevented ethanol-induced cell death in a dose-dependent manner. Interestingly, the formulation BV-7310 showed synergistic activity than any individual extract tested in this assay. BV-7310 also showed potent antioxidant activity in 2,2-diphenyl-1-picryl-hydrazyl (DPPH) assay. Next, we induced hepatitis in Sprague–Dawley (SD) rats using repeated alcohol (40%) dosing, and carbon tetrachloride (CCl_4_) 24 hours before termination. Both oral doses of BV-7310 (250 and 500 mg/kg body weight) protected the alcohol-induced body weight loss and significantly improved the elevated levels of liver enzymes compared to the vehicle treated group. Thus, BV-7310 prevents alcohol-induced toxicity in both in-vitro and in-vivo models and could be beneficial for the treatment of ALD or other conditions, which may cause liver toxicity.

## 1. Introduction

Liver is the major organ involved in the metabolic disposal of ethanol. Cytosolic alcohol dehydrogenase, microsomal ethanol-oxidizing system, and peroxisomal catalase metabolize ethanol (EtOH) to acetaldehyde [1]. The latter is a reactive metabolite that can produce injury in a variety of ways. Acetaldehyde is further metabolized to acetate by acetaldehyde dehydrogenase, which is abundant in liver mitochondria. Since the rate of acetaldehyde formation is also highest in the liver, it is one of the early targets for alcohol-induced injury. Nonoxidative pathways of ethanol metabolism affect mitochondrial function by forming ethyl esters of long chain fatty acids. Carbon tetrachloride (CCl_4_) is a halogenated xenobiotic compound that was earlier used as a dry-cleaning solvent and a refrigerant in fire extinguishers. Currently its industrial use is stopped due to well-documented adverse health effects. Exposure to CCl_4_ results in centrilobular hepatic necrosis. Ethanol and CCl_4_ are both metabolized by cytochrome P450 2E1 (CYP2E1) to a highly reactive free radical, which in turn causes hepatotoxicity through lipid peroxidation [2–5].

In view of the pathogenesis and increasing incidence of alcohol-induced liver disease, there is a need for screening for potential hepatoprotective agents. In this context, several candidate molecules like S-adenosyl methionine [6], catechin [7], propylthiouracil [8, 9], colchicines [10], and silymarin [11] have been already tested. However, their toxicity profiles, costs, and insignificant clinical and biochemical efficacy are some of the limiting factors, and there is a definite need and paucity of effective and affordable therapies for prevention and management of alcoholic liver disease [12].

As conventional medicine pursues a more integrated approach for disease management, herbs that influence liver efficiency are being revisited and evaluated for their overall health promoting effects. The science of Ayurveda offers a host of herbs and new phytopharmaceutical products that can be used to manage a spectrum of liver related imbalances [13–17]. While dealing with liver problems, the primary goal in Ayurveda is to enhance its detoxification processes thus preventing further damage [18].

BV-7310 is a combination of standardized extracts of four herbs. Whole-dried plants of *Phyllanthus niruri* (Bhuiamla) and *Andrographis paniculata* (Kalmegh), along with roots of *Tephrosia purpurea* (Sharapunkha) and *Boerhavia diffusa* (Punarnava), were used. All four herbs individually, or in combination of one or two, are being used in Ayurveda for several hundred years for gastrointestinal, liver, spleen, and renal disorders [19–21]. To our knowledge, this is the first combination of these four plants. We attempted to test this novel combination in alcohol-induced toxicity in cells and in animals. The plant samples in these studies were accessed under an authorization by the National Biodiversity Authority of the Government of India. The individual plant components of BV-7310 were identified, authenticated, deposited, and registered at the Herbarium of Central Council for Research in Ayurvedic Sciences, (CCRAS), Department of Ayush, Government of India. The herbarium voucher numbers are *A. paniculata (4401), B. diffusa(4402), T. purpurea (4403), and P. niruri (4404*).

## 2. Materials and Methods

### 2.1. Preparation and Standardization of Herbal Extracts

The raw materials or whole plants used in the formulation were procured from Ambadas Vanaushadhalaya, Pune, India, and were authenticated and standardized to ensure reproducibility of results. The plants were extracted by a proprietary and US patented method [22] to give the most optimal yield of the medicinal properties or bioactive contents. Briefly, the method consists of presoaking the plant materials simultaneously in aqueous alcohol and another organic solvent like chloroform, and then defatting with hexane. Each of the individual plants and final formulation BV-7310 was standardized using thin layer chromatography (TLC) and high-performance liquid chromatography (HPLC) and subjected to stringent stability tests so that the efficacy and safety of the finished formulation is reproducible. Certificate of analysis was compared every time to see batch to batch variation, if any. The dry powders were mixed in a weight/weight ratio of *P. niruri :T. purpurea : B. diffusa : A. paniculata :* 2.5 : 1.75 : 1.5 : 1, respectively, to formulate BV-7310.

### 2.2. Thin-Layer Chromatography (TLC) and High-Performance Liquid Chromatography (HPLC) of Extracts and Identification of Bioactive Ingredients

Chromatographic separation was achieved on TLC plates (20 × 20 cm) and precoated with silica gel 60 F254 (Merck, India) of 0.2 mm thickness. Samples were spotted using CAMAG Linomat and Sample Spotter (Camag, Switzerland) equipped with a 100 *μ*L syringe. Plates were developed in a glass chamber, and the experimental conditions were maintained at 20° ± 5°C.

*A. paniculata* and *B. diffusa* were run in dichloromethane : hexane : methanol (7 : 2 : 1) solvent system, *P. niruri* by dichloromethane : acetic acid : methanol (12 : 0.5 : 2) system, and *T. purpurea* by acetone : petroleum ether (4 : 6) system to identify the extent of bioactive compounds along with commercial standard marker compounds. Pure Andrographolide, Boeravinone B, Phyllanthin, and Rutin were procured from Sigma-Aldrich, USA. The TLC of the marker compounds showed major single spots, whereas the extracts and formulation showed many additional spots with all different Rf values. They were further standardized and characterized by HPLC along with standard commercial markers of the respective bioactive molecules. HPLC system and C-18 columns were from Waters Inc, USA. Methanol: water and acetonitrile: water were used to run the systems, respectively. All solvents were analytical grade and procured from Merck, India.

### 2.3. Cytotoxicity Assay of BV-7310 in Human Liver HepG2 Cells by 3-(4,5-dimethylthiazol-2-yl)-2,5-diphenyltetrazolium Bromide (MTT) Method

Human liver HepG2 cells were procured from the American Tissue Cell Culture (ATCC :HB-8065). They were cultured in 96-well plates (Nunc Technologies, USA) and grown to confluence in minimal essential medium (MEM) supplemented with 5% fetal bovine serum (FBS) and 1% of an Antibiotic-Antimycotic solution containing 100 units/ml of penicillin *G*, 100 mg/ml of streptomycin sulphate and 2.5 *μ*g/ml of amphotericin B. All solutions were procured from Gibco, USA. The following day, the growth medium was removed and replaced with growth medium containing BV-7310 at four different doses (0.5, 1.0, 10, and 50 *μ*g/ml), and a known cytotoxic drug Tamoxifen (Sigma-Aldrich) at 20 *μ*M final concentration in 0.1% DMSO, in quadruplicate wells per concentration. All stock solutions of the extracts were first made 1,000-fold excess in 100% DMSO, and further diluted in the media to achieve the final concentration of 0.1% DMSO. After 48 hours, the MTT assay was carried out per standard protocol to see the live cells. The MTT (Sigma-Aldrich, USA) assay protocol is based on the conversion of water soluble MTT compound to an insoluble formazan product. Fresh MTT was prepared at 1 mg/ml in growth media. Treatment media were removed and replaced with 200 *μ*l of MTT media. Plates were incubated for another 2 hours at 37°C and 5% CO_2_, then media were removed and replaced with 200 *μ*l of 100% ethanol to dissolve the formazan. Absorbance was read at 570 nm using a microplate reader (Bio-Rad, USA).

### 2.4. Ethanol-Induced Toxicity in HepG2 Cells and Effect of BV-7310 and Individual Plant Extracts

HepG2 cell lines were maintained as described before. In this assay, the cells were plated in 96-well plates and incubated overnight at 37°C in a CO_2_ incubator. Then the cells were challenged with 120 mM pure ethanol (Merck) diluted in DMEM, along with various concentrations (15, 50, and 100 *μ*g/ml in 0.1% DMSO) of BV-7310 or the medium alone (as normal control), for 48 hours, in an incubator at 37°C with 5% CO_2_. At the end of 48 hours, alcohol-induced cytotoxicity was assessed by estimating the viability of the HepG2 cells by sulforhodamine (SRB) assay (Sigma-Aldrich, USA) as described earlier [23]. The SRB assay was used for cell density determination, based on the measurement of cellular protein content with slight modifications which was used in the 96-well format. After the incubation period, cell monolayers were fixed with 10% (wt/vol) trichloroacetic acid (Merck, India) and stained for 30 min, after which the excess dye was removed by washing repeatedly with 1% (vol/vol) acetic acid. The protein-bound dye was dissolved in 10 mM Tris (pH 8.0) solution for determination at 510 nm using a microplate reader.

In another set of experiments, we compared the efficacy of all four individual plant extracts at a dose of 50 *μ*g/ml each versus the final formulation, BV-7310 at a dose of 50 *μ*g/ml. Cells were first plated for 24 hours in 96-well plates and then challenged with 120 mM ethanol along with each of the individual plant extracts and BV-7310. Some cells were treated only with DMSO as vehicle control cells. After 48 hours of incubation at 37°C in the CO_2_ incubator, toxicity was measured by the SRB method as described before.

### 2.5. Evaluation of Antioxidant Activity

The free-radical scavenging activity of individual extracts and BV-7310 formulation was analyzed using 2,2-diphenyl-1-picryl-hydrazyl (DPPH), following a standard protocol with slight modification [24]. DPPH (0.1 mM) solution was freshly prepared in methanol (39.4 mg in 1,000 ml). Different concentrations of extracts were added at an equal volume (3 ml) to DPPH solution. The mixtures were shaken vigorously and allowed to stand at room temperature for 30 minutes. Then the absorbance was measured at 517 nm using a UV-VIS spectrophotometer (Agilent Technologies, USA). Ascorbic acid (Sigma-Aldrich, USA) was used as the reference. All the tests were performed in triplicates, and the results were averaged. The IC_50_ values were calculated using linear regression analysis and used to indicate antioxidant activity.

### 2.6. Daily Oral Alcohol Dosing Followed by Acute Carbon Tetrachloride-Induced Hepatitis in SD Rats

The aim of this study was to see the effect of BV-7310 in rats after repeat dosing of 40% oral alcohol, followed by a single subcutaneous injection of CCl_4_ (Merck) at a dose of 0.1 ml/kg body weight with liquid paraffin 1 : 1 [25, 26]. The animal study was conducted in the most humane manner, and all possible steps were taken to avoid animal suffering at every stage of the experiment. At the end of the study, the animals were euthanized under appropriate anesthesia. The study was conducted as per Institutional Animal Care and Use Committee (IACUC) standards, and in accordance with the internationally accepted principles for laboratory animal use and care as directed by the Organization for Economic Co-operation and Development (OECD), Principles of Good Laboratory Practices (GLP), European Economic Community (EEC), and the United States Food and Drug Administration, Title 21 CFR. The study was approved and monitored by an appropriate in-house Ethics Committee.

A total of 48 SD rats (24 males and 24 females) of approximately 12–15 weeks old and weighing between 120 and 200 gm were reared at Bioved Pharmaceuticals Pvt. Ltd., Pune, India. The sterilized husk and bedding were supplied by Prashant Traders, Pune, India. The animals were acclimatized for seven days before starting the experiment. They were in 12 hours light and dark cycle, with purified water ad libitum and regular rodent diet from Nav Maharashtra Chakan Oil Mills, Pune. Three rats of the same sex were housed in a single cage. Rats were divided into four groups of 12 animals, each containing 6 Males and 6 Females. Group I was sham control where no alcohol or BV-7310 was given, and they received only purified water and food by oral route for 21 days. Group II received only 40% ethanol (Merck, India) (V/V, 2.0 ml/100 gm body weight) in distilled water for 21 days, and CCl_4_ (Merck, India) subcutaneously at a dose of 0.1 ml/kg body weight with liquid paraffin 1 : 1, on the 20^th^ day. Groups III and IV received BV-7310 daily at doses of 250 mg/kg and 500 mg/kg body weight, respectively, along with ethanol for 21 days and CCl_4_ on the 20^th^ day. Thus, liver damage was induced by repeat doses of 40% ethanol plus an acute subcutaneous insult of CCl_4_ sufficient to potentiate the damage [26]. Twenty-four hours after the last oral dose, on Day 21, rats were anesthetized with diethyl ether, and blood was collected for clinical chemistry parameters. Serum levels of total protein, alanine aminotransferase (ALT), aspartate aminotransferase (AST), alkaline phosphatase (ALP), and bilirubin were determined using the Erba Chem-5 Plus Selective Multi Parametric Clinical Chemistry Analyzer (Transasia Inc.).

### 2.7. Statistical Analysis

The data are expressed as mean ± SEM. Statistical analysis was made by one-way ANOVA followed by Tukey–Kramer multiple comparison tests; *p* values ≤ 0.05 are considered as significant.

## 3. Results

### 3.1. Identification and Characterization of Bioactive Components in the Extracts

**Figure 1:**
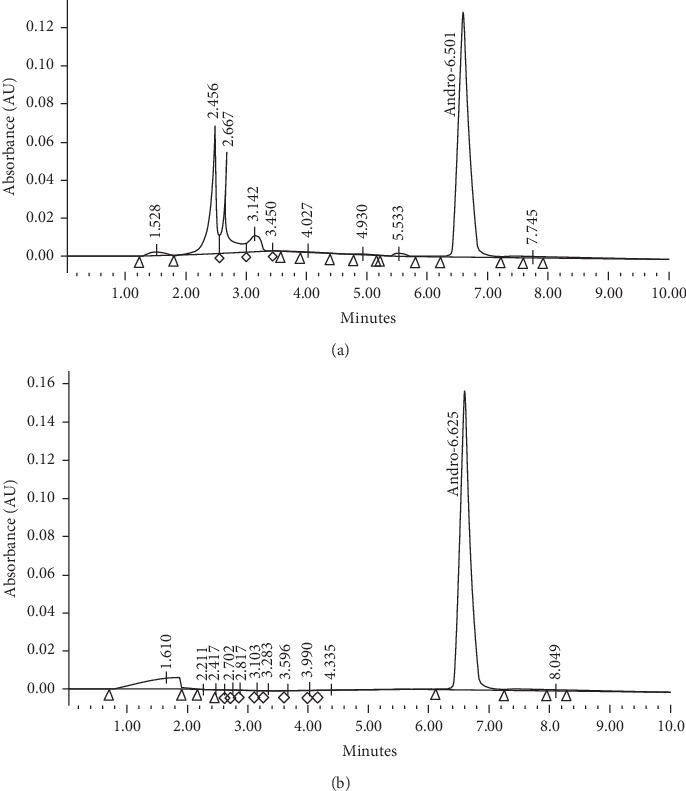
BV-7310 *Andrographis paniculata* extract and standard Andrographolide HPLC profile. (a) *Andrographis paniculata* extract in BV-7310. (b) Standard Andrographolide.

Pure compounds like Andrographolide from *A. paniculata*, Boeravinone B from *B. diffusa*, Phyllanthin from *P. niruri,* and Rutin from *T. purpurea* have shown to have antioxidative stress, antilipid peroxidation, steatosis, or antiviral effect in both in-vitro cellular and in-vivo rodent models of hepatotoxicity by a number of pathways including PPARγ, SREBP1, NF*κ*B/TNF-*α*, HO-1, or IL6/STAT-3 pathway [27, 28]. As mentioned earlier, all four herbs were extracted by a novel, proprietary, and patented method to yield a unique enriched component or fraction of each plant [22] as analyzed by HPLC. As an example, pure commercial Andrographolide was analyzed and observed at a retention time of 6.9 min (Figure 1(a)). In the same solvent system, the proprietary *A. paniculata* extract gave a HPLC chromatogram (Figure 1(b)) that was enriched in Andrographolide at same retention time and showed other peaks. Similarly commercial Boeravinone B from *B. diffusa* extract, Phyllanthin from *P. nirruri* extract, and Rutin from *T. purpurea extract* showed matching similarities in retention time of the major active components (data not shown). The minor peaks in every preparation may have some additional beneficial effect on prevention of toxicity and/or in potentiating the efficacy.

### 3.2. Cytotoxicity of BV-7310 in HepG2 Cells

**Figure 2:**
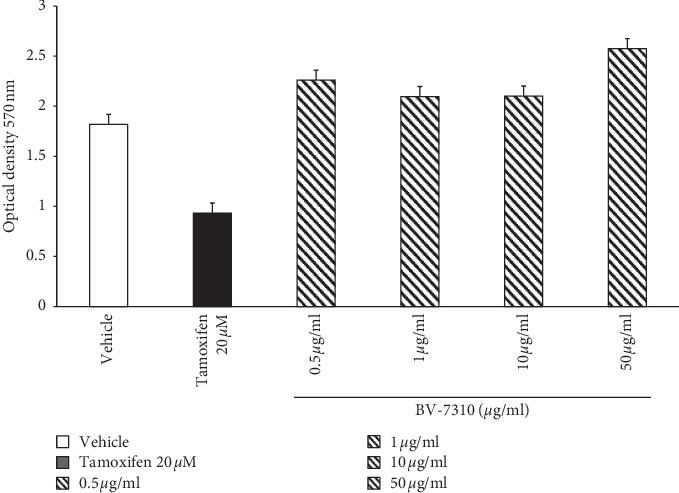
Cytotoxicity of BV-7310 in HepG2 cells by MTT Method.

To assess the toxicity levels of this unique four plant mixture, first we exposed this formulation to human liver HepG2 cells for 48 hours along with a cytotoxic drug tamoxifen as positive control. Within 48 hours of incubation, 20 *μ*M of tamoxifen killed almost 50% of cells. Optical Density (OD) at 570 nm represents the number of live cells that metabolize the MTT to form formazan, whereas BV-7310 at all doses (0.5, 1.0, 10.0, and 50.0 *μ*g/ml) did not show any toxicity to these HepG2 cells (Figure 2). The colorimetric procedure to see cellular metabolic activity like NAD(P)H-dependent cellular oxidoreductase enzymes may reflect the number of live cells present in the wells, and it is very clear, even at a dose of 50 *μ*g/ml for 48 hours, there is no cytotoxicity with BV-7310.

### 3.3. BV-7310 Protects HepG2 Cells from Ethanol-Induced Toxicity

**Figure 3:**
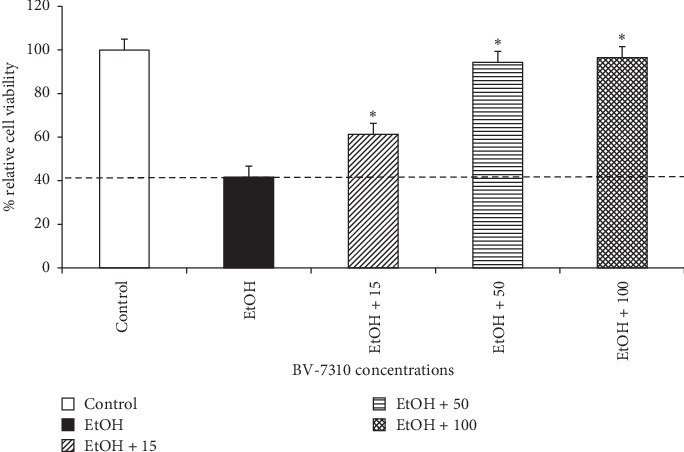
Dose response curve of BV-7310 on ethanol-induced toxicity in HepG2 cells. ^*∗*^Statistically significant *p* ≤ 0.05 against ethanol-treated cells.

At first, we treated the cells at different concentrations of ethanol (EtOH) and established a dose response curve at 48 hours (data not shown). At 120 mM ethanol concentration, cell viability was around 40–42% as measured by sulforhodamine B (SRB) assay. When the cells were coincubated with BV-7310 (three different doses) along with 120 mM ethanol, the cell death was diminished significantly (Figure 3). BV-7310 even at 15 *μ*g/ml showed statistically significant inhibition of cell death, and dose dependently protected the ethanol-induced cell death in HepG2 cells. The dotted line on Figure 3 indicates the alcohol-induced cell death. All values above the line show cell protection in presence of BV-7310.

**Figure 4:**
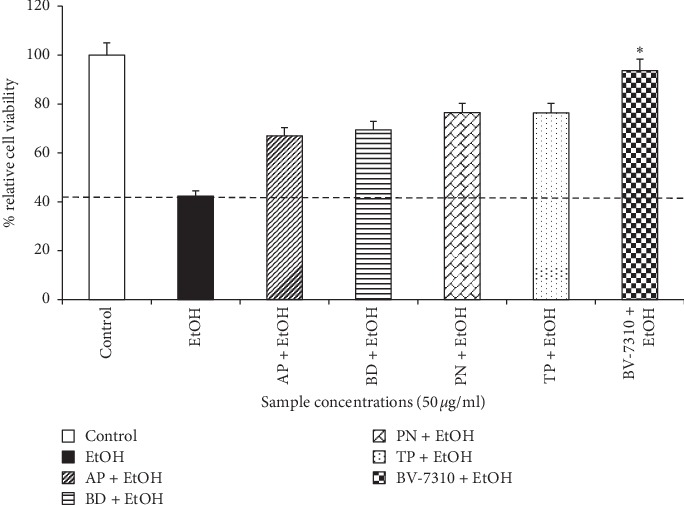
BV-7310 protects ethanol-induced HepG2 cell death better than individual plant extracts. ^*∗*^Statistically significant *p* ≤ 0.05 against ethanol-treated cells.

When all four individual plants were compared with BV-7310 formulation at the same dose level (50 *μ*g/ml) in the same ethanol-induced toxicity assay in HepG2 cells, BV-7310 showed higher protection than any single individual plant tested (Figure 4). Furthermore, the total amount of individual extracts exposed per cell in the combination BV-7310 is significantly less than the individual plant extracts of 50 *μ*g/ml. This indicates a possible synergistic activity of the four plants to attenuate the damages caused by alcohol, by affecting multiple pathways or different signalling events upon alcohol-induced toxicity. The dotted line in Figure 4 indicates the alcohol-induced cell death. All values above the line show cell protection. Interestingly, in other studies, 50 *μ*g/ml *Tephrosia* and *Phyllanthus* extracts individually have also shown significant protection on alcohol-induced damage to these cells [27, 28].

### 3.4. DPPH Antioxidant Activity of BV-7310

**Figure 5:**
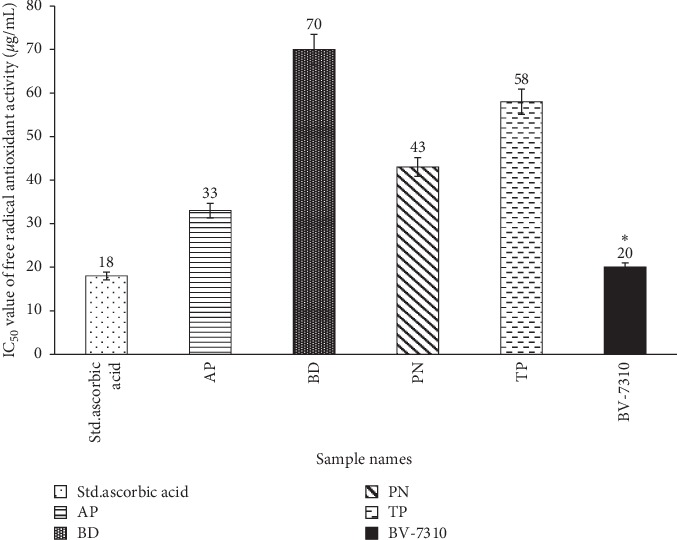
Antioxidant activity by DPPH activity—IC_50_ values of standard ascorbic acid, individual plant extracts, and BV-7310. ^*∗*^Statistically significant *p* ≤ 0.05.

The DPPH assay measured the relative antioxidant ability of individual plant extracts, BV-7310, and ascorbic acid as control. In our study, 50% inhibitory concentration (IC_50_) of ascorbic acid was 18 *µ*g/ml. Individual extracts showed IC_50_ of *A. paniculata* 33 *μ*g/ml, *B. diffusa* 70 *μ*g/ml, *P nirur*i 43 *μ*g/ml, and *T. pupurea* 58 *μ*g/ml. The formulation BV-7310 showed an IC_50_ of 20 *μ*g/ml, which is lower than any individual extract, as shown in Figure 5.

### 3.5. BV-7310 Protects Alcohol-Induced Body Weight Loss and Liver Damage in SD Rats

**Figure 6:**
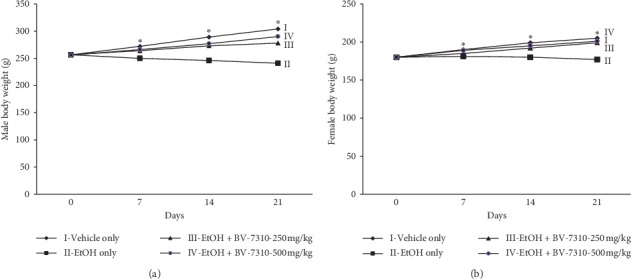
BV-7310 restores body weight loss upon oral dosing of ethanol. ^*∗*^Statistically significant *p* ≤ 0.05 against ethanol-treated group.

In this model, BV-7310 was given along with ethanol to rats for 20 days. Within 7 days, there was a significant loss of body weight in alcohol-treated rats compared to sham control. Animals given both the BV-7310 doses, 250 mg/kg (Group III) and 500 mg/kg (Group IV), in both sexes, showed significantly improved body weight loss compared to alcohol only group (Figure 6(b) in male rats and 6B in female rats). This effect on body weight was evident on Day 14 and Day 21. This clearly shows that BV-7310 prevented body weight loss, either by helping the anorectic effect of alcohol, or by counteracting the toxic effects of alcohol on overall metabolism as described previously [29].

The xenobiotic compound, carbon tetrachloride was given 24 hours before blood collection and did not have any effect on body weight, but the serum biochemistry parameters were altered significantly as shown in Table 1.

In both sexes, upon this alcohol-CCl_4_ treatment, AST increased by almost 4 times and ALT almost doubled compared to the sham control group. Total protein remained very similar in all the groups. However, in this dual insult model, BV-7310 showed a marked normalizing effect on serum transaminases, alkaline phosphatase, and serum bilirubin values as compared to alcohol-treated group. It is noteworthy to mention here, both acute toxicity in mice and long-term toxicity (180 days) in rats have proven that the formulation is safe for consumption even at doses 5–10 times higher than the prescribed therapeutic dose (manuscript in preparation).

## 4. Discussion

Excessive alcohol consumption is associated with the appearance of excess fat in the parenchymal cells. More complex acute changes include the appearance of intracellular hyalin and an acute inflammation, with finally a fibrotic hepatic disease. Alcoholic liver disorders comprise of fatty liver, hepatitis, and cirrhosis that are observed either alone or in combination. Malnutrition, genetic variations, or improper immunological mechanisms may act along with alcohol in causing liver injury. Daily drinkers are more susceptible to harmful effects than occasional drinkers. The liver processes most of the xenobiotic compounds, industrial chemicals, and prescription, or over-the-counter (OTC) drugs. It is the liver that gets the highest exposure of these toxic substances and their metabolites. There is no known drug to alleviate this alcohol- or chemical-induced liver damage. There are few plants or herbal formulations claimed to have some effect but not widely accepted [15, 16]. Hence, a safe drug or herbal preparation is long needed to address these issues.

BV-7310, an herbal preparation, contains extracts of medicinal plants specifically indicated for the treatment of gastrointestinal, liver, spleen, and renal disorders. Human liver HepG2 cells have served as a good model to study the hepatotoxicity of different chemicals or drugs, as these cells retain many of the morphological and biochemical characteristics of normal hepatocytes. We hypothesize that the combination of these four plants affects a multitude of different and distinct pathways of alcohol- and/or CCl_4_-induced liver damage, by managing the reactive free radicals to down-regulate pro-inflammatory pathways. That is the reason this novel formulation worked very well versus any of the individual extracts. It is interesting to see *P. niruri* extract alone can alleviate some liver injury and other ailments. It is present in highest percentage in our formulation [17, 19]. In contrast, Liv-52, a well-known liver product showed some down regulation of cytokines and PPARγ mediated pathway in HepG2 cells, but some clinical studies have questioned its true benefits [30–32].

Prolonged exposure to alcohol develops fatty changes in the liver of rats. However, unlike humans, they do not exhibit more severe forms of liver injuries such as hepatitis and cirrhosis. Hence, to exacerbate the pathophysiological conditions of the disease, CCl_4_ was included in the study. The alcohol-treated group lost significant body weight in a couple of days and recovered in a dose-dependent fashion in both groups on BV-7310. Addition of an acute dose of CCl_4_ aggravated the liver enzymes, and the formulation improved all these biochemical parameters at statistically significant levels. Further studies may be necessary to dissect out the alcohol only effect on these liver enzymes.

The data presented here suggest a strong hepatoprotective effect of this new formulation, which protected the ethanol-induced damage in human hepatoma cells compared to any of the single plants alone. It significantly reduced the levels of ALT, AST, ALP, and bilirubin upon several days of ethanol treatment followed by an acute CCl_4_ insult. This suggests that BV-7310 could be used as a new hepatoprotective formulation for alcohol- and/or drug-induced toxicity. More mechanistic work and acute and chronic toxicity studies along with controlled clinical trials are necessary to substantiate these claims.

## Figures and Tables

**Table 1:** Blood chemistry of animals at the end of the study.

Groups	Total protein (g/dl)	Serum alanine transaminase ALT (IU/L)	Serum aspartate aminotransferase AST (IU/L)	Alkaline phosphatase ALP (IU/L)	Serum bilirubin (mg/dl)
Male rats					
I	6.32 ± 0.31	44.86 ± 6.93	116.77 ± 11.11	155.13 ± 10.35	0.51 ± 0.05
II	6.23 ± 0.25	171.90 ± 74.37	242.80 ± 73.59	215.15 ± 62.02	0.87 ± 0.11
III	6.24 ± 0.30	69.27 ± 11.8^*∗*^	151.07 ± 36.21^*∗*^	138.65 ± 15.83^*∗*^	0.59 ± 0.05^*∗*^
IV	6.46 ± 0.24	66.01 ± 9.8^*∗*^	153.77 ± 31.05^*∗*^	130.13 ± 8.85^*∗*^	0.54 ± 0.13^*∗*^

Female rats					
I	6.01 ± 0.27	40.92 ± 5.33	111.47 ± 12.99	118.27 ± 14.72	0.53 ± 0.06
II	6.03 ± 0.50	156.87 ± 26.13	352.05 ± 63.32	187.80 ± 27.83	0.87 ± 0.14
III	6.11 ± 0.07	49.24 ± 4.06^*∗*^	139.83 ± 26.21^*∗*^	139.48 ± 23.86^*∗*^	0.60 ± 0.08^*∗*^
IV	6.03 ± 0.11	68.08 ± 5.57^*∗*^	155.95 ± 26.53^*∗*^	137.10 ± 16.75^*∗*^	0.62 ± 0.12^*∗*^

^*p*^ value derived from values compared with group II (alcohol-treated group). ^*∗*^Statistically significant *p* < 0.05.

## Data Availability

All raw data underlying the text, tables, figures, and conclusions of this study are available from the corresponding author upon request.
